# Metallic hairpin inhalation: a healthcare problem facing young Muslim females

**DOI:** 10.1186/s40463-014-0021-y

**Published:** 2014-08-02

**Authors:** Nabil Rizk, Noor E Gwely, Vincent L Biron, Usama Hamza

**Affiliations:** 1 Clinical Lecturer of Surgery, Department of Surgery, Division of Otolaryngology – Head and Neck Surgery, 1E4.34 Walter Mackenzie Science Center, University of Alberta, Edmonton T6G 2B7, AB, Canada; 2 Otolaryngology Department, Mansoura University, Mansoura, Egypt; 3 Cardiothoracic Surgery Department, Mansoura University, Mansoura, Egypt

## Abstract

**Objectives:**

To perform an epidemiological assessment of metallic hairpin inhalation in young Muslim females and highlight the need for a health education program in this population.

**Methods:**

We performed a retrospective analysis of females with a history of metallic hairpin inhalation presenting to the Otolaryngology and Cardiothoracic Surgery Departments at Mansoura University Hospitals from January 2000 to October 2006.

**Results:**

A total of 83 patients were identified with metallic hairpin inhalation, of which 2 were excluded as they were coughed and expelled by the patient. Ages ranged from 7 to 19 years. A history of inhaled foreign body (FB) was found in all cases but the majority of patients were asymptomatic, with only 6 patients (7%) presenting with cough. Chest x-rays confirmed the presence of metallic hairpin inhalation in all cases. The metallic hairpins were present in the trachea in 7 patients (9%), in the left bronchial tree in 43 patients (53%) and in the right bronchial tree in 31 patients (38%). Rigid bronchoscopy was performed in all patients with a retrieval rate of 80%. Repeat bronchoscopy was performed in 16 patients (20%), which was successful in 11 patients (14%). The remaining 5 patients required thoracotomy for removal of the metallic hairpin (6%).

**Conclusion:**

The significant number of inhaled metallic hairpins in young Muslim females highlights the need for a health education program in this population. Rigid bronchoscopy remains the primary tool for retrieval of these inhaled foreign bodies. However, when repeat broncoscopy is necessitated, a thoracotomy may be required.

## Introduction

Inhaled foreign body may result in acute respiratory distress, atelectasis, sepsis or death [1]. In the United States, greater than 300 children per year may die as a result of foreign body inhalation, with the majority of cases occurring in boys.

The most commonly inhaled foreign bodies include food, coins, dentures and metallic objects. Rarer cases of inhaled foreign bodies reported include broken tracheostomy tubes, hypodermic needles and more recently, the metallic scarf needle used by Arabic women for traditional Hijab [2],[3]. These metallic hairpins are used extensively in Islamic regions for securing facial and head scarves. A common presenting history in metallic hairpin inhalation involves a girl holding a metallic hairpin with her lips or teeth while fixing her turban, in order to get free hands to adjust the scarf. Talking, laughing or coughing while fixing the scarf may result in inadvertent inhalation of the pin into the trachobronchial tree [4]. These pins have a long slim body and a round-colored plastic bead at one end. This beaded end is heavier than the rest of the pin and therefore the pin usually falls with the beaded end pointing downward.

The objective of this study is to describe a retrospective review of metallic foreign body inhalation in young Muslim females in Egypt. We describe the clinical presentations, diagnosis and management of this unique otolaryngologic problem.

## Methods

Through a retrospective chart review, we identified all patients with a history of metallic hairpin inhalation between January 2000 to October 2006, presenting to Otolaryngology and Cardiothoracic Surgery Departments of Mansura University Hospitals. Data was obtained in accordance with the Mansura University Hospitals health research ethics board. Demographic data was collected including age, gender and place of residence. We reviewed charts to identify presenting symptoms, chest x-ray findings and management plans such as bronchoscopy and thoracotomy. These data were calculated to determine the percentage of foreign body identified in specified locations, the rates of successful bronchoscopy and need to thoracotomy.

Most of the patients were admitted to hospital early (within the first 24 hours). Rigid bronchoscopy was done in all patients in an operating room at emergency department under general anesthesia. In cases where repeat endoscopy was necessary a senior surgeon perfomed the procedure. Patients’ families were consented for the possible need for thoracotomy in case of second bronchoscopy failure. A trial of fiberoptic retrieval was done in selected cases. Thoracotomy and bronchotomy was done for removal of the FB when other measures failed during the second bronchoscopy.

## Results

A total of 83 female patients were identified with a diagnosis of metallic hairpin inhalation. Two patients self-retrieved the FB by coughing and were therefore excluded from analysis. Of the 81 patients analyzed, the presenting age ranged from 7 to 19 years, with mean and median ages of 13.42 years and 13 years, respectively. No patients were found to have any neurological impairments, pulmonary diseases or other illnesses predisposing them to aspiration.

**Figure 1:**
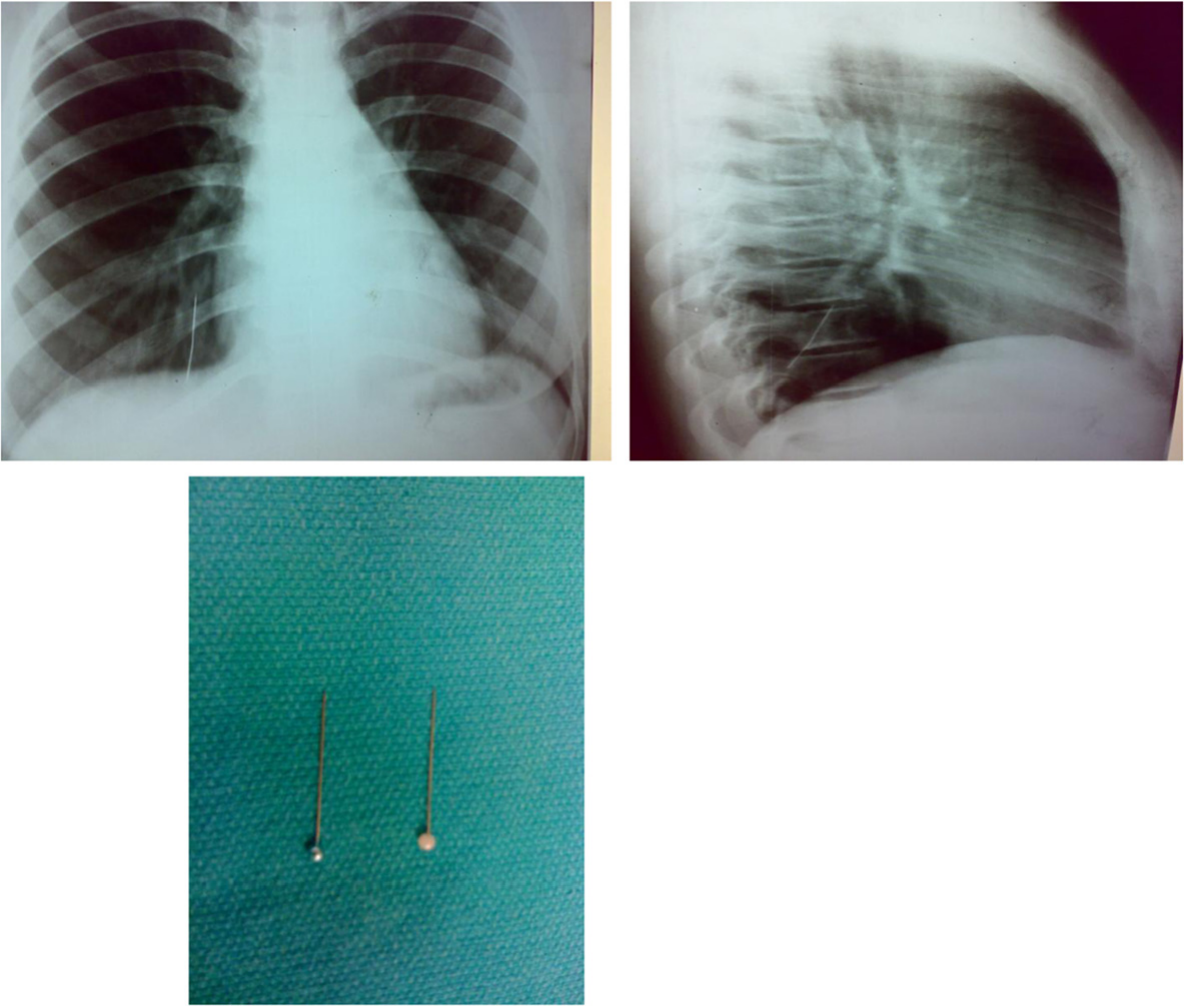
Posterio-anterior (left) and laterl (right) X-ray of a patients who inhaled a metallic hair pin (bottom).

Presentation was early (within the first 24 hours) in 95% of patients. Late presentation occurred in 5% of patients (24 hours to 15 days). Most patients with late presentations resided in a rural area. All patients gave a history of FB inhalation in the form of choking or coughing. At presentation to the emergency department, 6 patients (7%) complained of cough but all others were asymptomatic. There was no specific physical examination finding, as this was a non-obstructing foreign body aspiration. Postero-anterior and lateral chest X-rays documented the presence of FB in all cases (Figure 1). There was no radiological evidence of obstructive changes in any of these patients.

As shown in Table 1, the FB was present in the trachea in 7 (9%) patients, the left bronchial tree in 43 (53%) of patients (18 in main bronchus and 25 in segmental and sub-segmental bronchi) and in the right bronchial tree in 31 (38%) patients (14 in main bronchus and 17 in segmental and sub-segmental bronchi). Rigid bronchoscopy was performed in all patients under general anesthesia with an 80% success rate.

**Table 1:** Patient characteristics, symptoms and location of foreign body

**Data**	**Number (percentage)**	p-value*
History of FB	83 (100)	
Presentation		
Early	79 (95)	
Late	4 (5)	
Symptoms at presentation	6 (7)	
Location of 81 foreign bodies	81	
1-Tracheal	7 (9)	<0.001
2-Lt Bronchial tree	43 (53)	<0.001
A-Main bronchus	18 (42)	
B-Segmental and sub-segmental	25 (58)	
3-Rt Bronchial tree	31 (38)	<0.001
A-Main bronchus	14 (46)	
B-Segmental and sub-segmental	17 (54)	

*p-value determined using chi-square test assuming an expected equal distribution of locations for 1, 2 and 3.

Chest x-rays were done prior to repeat bronchoscopy, which documented distal migration of the FB in 16 (20%) of patients. Repeat bronchoscopy was successful in 11 (14%) patients [rigid bronchoscopy in 8 (10%) and fiberoptic bronchoscopy (FOB) in 3 (4%)].

Post procedure chest x-rays revealed no complications related to bronchoscopy. Thoracotomy and bronchotomy for removal of the FB was done in 5 (6%) of patients (Table 2). In all of these patients, the FB was lodged in sub-segmental bronchi. Length of hospital stay for patients that underwent brochoscopic removal was one day and was 5-8 days for patients who underwent a thoracotomy.

**Table 2:** Procedures done for removal of foreign body

**Procedure**	**Number of patients (percentage)**
Bronchoscopic retrieval	81 (100)
Successful removal by rigid bronchoscopy	65 (80)
Trial removal by redo bronchoscopy	16 (20)
A- RB (Rigid bronchoscopy)	8 (10)
B-FOB (Fiberoptic bronchoscopy)	3 (4)
Successful removal by redo broncoscopy	11 (14)
Failed bronchoscopy	5 (6)
Thoracotomy	5 (6)

## Discussion

The aspiration of foreign bodies is becoming a common problem in tertiary referral hospitals in Islamic nations. The common age group for tracheobronchial foreign body aspiration is between 6 months and 4 years with a male predominance [5]. In adults, inhaled FB is unusual and is associated with decreased level of consciousness, neuromuscular abnormalities and alcohol consumption. Many authors reported the scarf metallic pin inhalation from Egypt, Jordan and Turkey, which demonstrate some differences as it occurs in adolescent girls.

In our study, the median age was at presentation was 13 years. Only 8 (10%) of girls were below 10 years old. In Islamic countries, females begin to wear scarves when secondary sex characteristics appear. Wearing a turban and attaching pins properly is a very complex task for young females. They are less attentive than older adolescents and adults, therefore, with delicate maneuvers a loss of concentration, this can lead to FB aspiration. Our review of presenting histories highlights strikingly similar scenarios leading to metallic hairpin inhalation. The aspiration of these pins occurs while talking, laughing and coughing while attempting to secure their turban [6].

It is commonly believed that inhaled foreign bodies are lodged preferentially in the right bronchial tree due to its more direct route. However, in our study, the metallic hairpin was more commonly lodged in the left bronchial tree (53%), consistent with other reports. Al-Halfawy [2] reported that 19 out of the 32 pins (59.4%) were in the left bronchial tree. Al-Lawaty et al. [7] showed that in a series 39 retrieved pins, 22 (56.4%) were in the left bronchial tree. Rageb et al. also reported that inhaled metallic pins were more often lodged in left bronchial tree than the right side with statistical significance. They attributed this finding to the Bernoulli phenomenon. Coughing, laughing or speaking creates negative pressure. The relatively narrower diameter of the left bronchus compared to the right one creates preferential negative pressure in the left bronchial tree. Taken together, the Bernoulli effect of the left bronchus appears to outweigh the anatomically vertically positioned right bronchus in the case of metallic hairpin inhalation.

In our series of metallic hairpin inhalation cases, patients did not have clinical or radiological signs of obstruction and therefore most patients were asymptomatic. However, an infection or mild granulation around the pin may cause some discomfort in late presentation. The major symptom reported in the literature is coughing [8], consistent with our findings.

Location of the pins, the physician who is performing the bronchoscopy, early admission and number of interventions, are important factors that influence morbidity. In this patient series, the first bronchoscopic evaluation was performed by an experienced otolaryngologist. However, distally located pins could not always be extracted by a rigid bronchoscope. During rigid bronchoscopy, the pointed end of the turban pin should be grasped and extracted through the bronchoscope. The pointed end can harm the bronchial wall if the turban pin is grasped and pulled from the other part of the pin. There are reports in the literature [9]–[12] describing foreign bodies that could not be removed by bronchoscopy due to their distal location.

Our thoracotomy rate was 6% in 81 patients presented with metallic pin inhalation. Kaptanoglu et al. [4] reported a thoracotomy rate of 1.6% in their patients and Ucan et al. [13] reported a thoracotomy rate of 4%. Possible causes of higher incidence of thoracotomy in our patients were the distal location of FB at sub-segmental bronchi and late presentation after the onset of aspiration. Moreover, some patients had been referred to us from other centers, following unsuccessful bronchoscopies.

## Conclusions

Metallic hairpin inhalation is becoming an increasingly common problem in Islamic countries. We also believe that the public should be made aware of this problem through education and health care programs in school and media Alternative safer dressing methods (adhesive strips and snap fasteners) should be preferred. We should encourage young females to wear simple turbans that do not require metallic pin fixation.

## Competing interest

The authors declare that they have no competing interests.

## Authors’ contributions

NR, NG and UH participated in data collection, data analysis and writing of the manuscript. VB participated in writing the manuscript. All authors read and approved the final manuscript.
